# Worth the Risk? Greater Acceptance of Instrumental Harm Befalling Men than Women

**DOI:** 10.1007/s10508-023-02571-0

**Published:** 2023-03-17

**Authors:** Maja Graso, Tania Reynolds, Karl Aquino

**Affiliations:** 1 Faculty of Behavioural and Social Sciences, Department of Organisational Psychology, University of Groningen, Grote Kruisstraat 2/1, 9712 TS Groningen, The Netherlands; 2 Department of Psychology, University of New Mexico, Albuquerque, NM USA; 3 Marketing and Behavioural Science Division, Sauder School of Business University of British Columbia, Vancouver, British Columbia Canada

**Keywords:** Instrumental harm, Sacrificial harm endorsement, Gender, Sacrifice

## Abstract

Scientific and organizational interventions often involve trade-offs whereby they benefit some but entail costs to others (i.e., instrumental harm; IH). We hypothesized that the *gender* of the persons incurring those costs would influence intervention endorsement, such that people would more readily support interventions inflicting IH onto men than onto women. We also hypothesized that women would exhibit greater asymmetries in their acceptance of IH to men versus women. Three experimental studies (two pre-registered) tested these hypotheses. Studies 1 and 2 granted support for these predictions using a variety of interventions and contexts. Study 3 tested a possible boundary condition of these asymmetries using contexts in which women have traditionally been expected to sacrifice more than men: caring for infants, children, the elderly, and the ill. Even in these traditionally female contexts, participants still more readily accepted IH to men than women. Findings indicate people (especially women) are less willing to accept instrumental harm befalling women (vs. men). We discuss the theoretical and practical implications and limitations of our findings.

**Supplementary Information:**

The online version contains supplementary material available at 10.1007/s10508-023-02571-0.

## Introduction

The promise of achieving “the greater good” has inspired numerous interventions designed to move society toward presumably desirable ends. Companies develop and market products to improve quality of life, organizations introduce policies to improve employees’ workplace experiences, and educators implement practices to improve learning outcomes. These interventions are frequently justified by claims that the benefits to many outweigh the potential harms to a few—a moral argument consistent with a utilitarian ethical framework.^1^

A utilitarian approach to morality accepts inflicting harm onto some people if doing so increases the sum total of human happiness and well-being (e.g., Mill, 1861/2010; Singer, 1981, 2020). Guided by the classic tenets, Kahane et al. (2018) identified two elements that reflect the negative and positive features of utilitarian reasoning. The negative dimension—instrumental harm (colloquially known as collateral damage)—gives a moral agent permission to “instrumentally use, severely harm, or even kill innocent people to promote the greater good” (Kahane et al., 2018, p. 132). Impartial beneficence reflects the positive aspect of utilitarianism, requiring prioritization of the greater good above all else. In its purest form, this element demands people ignore personal ties, family loyalties, group memberships, special preferences, and emotional impulses that compromise impartiality and achieving this greater good (e.g., Hughes, 2017).

However, people frequently depart from such prescriptive moralities (Hughes, 2017; Kern & Chugh, 2009), seldom approaching the level of impartiality required to practice utilitarianism and accept sacrifices that may contribute to the greater good. Indeed, judgments about benefit and harm are highly subjective (Schein & Gray, 2018) and even malleable (Haslam, 2016; Rozin, 1999). This subjectivity, coupled with the difficulty of achieving consensus on what constitutes the *greater good*, undermines impartial calculi of costs and benefits requisite for upholding utilitarian principles.

The current investigation examined one factor that might compromise the impartial evaluation of social interventions: the gender of the person who experiences instrumental harm (Instrumental Harm). Based on prior research on perceptions of harm to women and men, we hypothesized that people asymmetrically support interventions inflicting collateral harm to men versus women. Such a bias violates the principle of impartial beneficence, potentially compromising the evidence-based advancement of men and women alike. As detailed below, our predictions are rooted in extant work on gender and moral decision-making and are extended to contexts depicting low-level harm.

### Asymmetry in IH Acceptance as a Function of Gender

Perhaps the most compelling test of utilitarianism’s impartial beneficence tenet is the trolley problem (Foot, 1978). In the classic version of this moral dilemma, individuals must consider whether they would save a few people tethered to trolley tracks by derailing the trolley to instead crush a single individual (also tethered). Impartial beneficence dictates this single individual be sacrificed for the greater good; personal characteristics of this unfortunate individual—including their gender—should be irrelevant to decision-making. Yet, in line with the bounded nature of moral judgment (Kern & Chugh, 2009), the sacrificial individual’s gender sways observers’ judgments. Indeed, FeldmanHall et al. (2016) demonstrated that when responding to this trolley problem, people were more willing to sacrifice a man than a woman. These patterns have been replicated using virtual reality (Skulmowski et al., 2014).

Although these findings suggest people more readily accept physical harm to men than women in life-versus-death contexts, it remains unclear whether these results translate to lower-level, but nonetheless consequential forms of harm (e.g., psychological, health, educational, sexual). Extant evidence provides some indirect support to this possibility: people perceive men as less physically vulnerable and report lower desires to help them than women (Burnstein et al., 1994; Dijker, 2001, 2010). These patterns tentatively suggest people should more readily accept various forms of instrumental costs borne by men than by women.

One explanation for these patterns is gender stereotyping. Gender is a social category linked to numerous stereotypes relevant to moral decisions about harm. Throughout history (Bem, 1974; Hoffman & Borders, 2001) and still today, gender stereotypes conceptualize men as aggressive, self-sufficient, and risk-accepting, and women as gentle, tender, and yielding (Bhatia & Bhatia, 2021; Donnelly & Twenge, 2017; Eagly et al., 2020; Ellemers, 2018; Lewis & Lupyan, 2020). These assumptions have been further differentiated into the domains of agency and warmth, wherein men are more closely linked to agency and women to interpersonal warmth (i.e., communion; Eagly et al., 2020; Fiske et al., 1999, 2007).

Eagly and Mladinic (1989, 1994) termed this divergence the “women are wonderful” effect, whereby women are regarded more positively due to their presumed communality, but men more negatively due to their boldness and relative lack of warmth. People espouse these beliefs implicitly, such that they more strongly dislike men due to their automatic associations between masculinity and potency to inflict destruction (e.g., rage-driven violence; Rudman et al., 2001). The stereotypes making women appear “wonderful” (Eagly & Mladinic, 1989; Glick et al., 2004) and communal have also been linked to people’s stronger inclination to perceive women as victims (Reynolds et al., 2020). Therefore, individuals may similarly apply a reflexive heuristic that women should be protected from harm, including even from the IH resulting from interventions potentially advancing a greater social good. In contrast, these harms will be viewed as more acceptable if borne by men. Accordingly, we test the following hypotheses:

#### Hypothesis 1

People will be more willing to endorse interventions when IH befalls men as opposed to women.

### Gender Differences in Partiality

Although we predict a greater tolerance for instrumental harm borne by men than by women, not all individuals will espouse such an asymmetry to equal degrees. A substantial body of evidence finds women exhibit stronger in-group biases favoring their own gender than do men (Rudman & Goodwin, 2004), suggesting greater acceptance of IH to men than women will be especially pronounced among women. Across countries, women express stronger hostility toward men and lower hostility toward women (Glick et al., 2004), suggesting if anyone should exhibit the hypothesized gender bias in instrumental harm acceptance, it should be women. Supporting this prediction, laboratory experiments find women redistribute payments to favor low-earning female (but not male) workers, whereas men showed no such gender bias (Cappelen et al., 2019). In the courtroom, women filing workplace discrimination claims were more likely to win compensation when their case was adjudicated by a female judge (Knepper, 2018). These patterns might be explained by a stronger bias in moral typecasting among women, whereby women more easily recognize other women as victims and men as perpetrators of harm (Reynolds et al., 2020). However, it remains unclear whether women show stronger gender biases in their tolerance of instrumental harms in low-level contexts. We predicted the following:

#### Hypothesis 2

Female participants will show a stronger asymmetry in their endorsement of IH, such that compared to male participants, female participants will show more approval of interventions inflicting instrumental harm onto men than onto other women.

### Boundary Contexts: Stereotypically Female Contexts

The same gender stereotypes contributing to the justification of women’s protection from harm (e.g., higher communality) may also highlight possible boundary contexts to Hypothesis 1. That is, the stronger tendency to protect women might disappear in contexts whereby gender stereotypes dictate that women should bear the costs of social progress, such as by sacrificing on behalf of infants, children, the elderly, and the infirm. If throughout history, women’s communal roles enhanced the well-being of vulnerable individuals (e.g., children and the elderly; Eagly & Wood, 1999; Geary, 2010), interventions benefitting those vulnerable individuals, but inflicting costs onto women, might be equally or more strongly tolerated than those inflicting harm onto men, who less often filled such caregiving roles. Indeed, people perceive women as more responsible than men for protecting their children from harm (Barry et al., 2020), suggesting that traditional gender roles contribute to perceptions of sacrificial obligations. Formally, we predicted:

#### Hypothesis 3

The bias to reject IH to women (Hypothesis 1)will be neutralized in caregiving domains whereby historically, women have been expected to sacrifice more than men.

### Present Research

We tested Hypotheses 1 and 2 across three complementary experimental studies (see Appendix Table 1 for an overview of all three studies). Study 1 provided the first test of our primary predictions in an organizational context. Study 2 utilized a broad array of contexts and interventions to test the generalizability of these patterns. Study 3 relied on stereotypical female caregiving contexts to provide a conservative test of the hypotheses and examine a potential boundary condition: domains wherein women have been traditionally expected to sacrifice (Hypothesis 3).

All studies were approved by the human subjects review boards from authors’ institutions. Participants provided their consent before they commenced the studies. Links to data and pre-registration documents (Study 1 and 3) are available at the end of this manuscript. We direct readers to supplementary online materials (SOM) for our materials, additional explanations, and exploratory analyses.

## Study 1: Assessment of Organizational Intervention

Participants evaluated a workplace intervention aimed at reducing mistreatment, which entailed IH to some employees. Many programs designed to improve the workplace have marked benefits, but some may entail negative and unintended consequences (Chang et al., 2019; Leslie, 2019; Singal, 2019). The reasons for failures are frequently traced to poorly designed or implemented initiatives (Janssens & Steyaert, 2019). However, some failures result from employees’ own interpretations or reactions to the programs. For example, some employees, particularly those from high-status groups, may react negatively to initiatives they perceive as threatening (Janssens & Steyaert, 2019; Lipman, 2018; Plaut et al., 2011). Study 1’s vignette drew from this research and a range of practitioner-oriented suggestions (e.g., Dobbin & Kalev, 2016; Lipman, 2018; Plaut et al., 2011; Zheng, 2019) to depict a toxic work environment and a plausible intervention designed to fix it (see SOM for full description). This study was pre-registered at https://aspredicted.org/blind.php?x=h67aq7.

## Method

### Participants

We aimed to retain at least 75 responses in each condition to achieve 80% power to detect an effect size of *r* = .21.^2^ Accordingly, we recruited 200 American individuals from Amazon’s CloudResearch (formerly TurkPrime; Litman et al., 2017) in exchange for $1.25 USD.

To assess engagement, we embedded 5 reading comprehension questions (true/false) and one attention check. We retained participants who missed no more than one check (additional information about our quality control process is available in SOM). Our final sample (*N* = 160) was 67.1% men. The average age was 34.5 years, SD = 9.7.

### Procedure

Using an online survey, participants evaluated the acceptability of an employee intervention program designed to improve toxic work environments (see Appendix Table 1 for a summary; see SOM for complete wording). We selected toxic behaviors that tend to be perpetrated by both men and women (Gabriel et al., 2017). The program reduced reports of general harassment, incivility, damaging gossip, and discrimination by 36%, thereby improving most employees’ workplace experiences. In a between-subjects design, participants were randomly assigned to one of two gender conditions wherein either male or female employees experienced IH in response to the program. Depending on the condition, either men or women found the program to be offensive and consequently experienced poor psychological outcomes. However, it is possible that participants perceived the instrumental harm of the program (i.e., feeling offended, poor psychological outcomes) as more severe when experienced by women versus men. To rule out the possibility that effects could be driven by perceptions that the negative externalities were perceived as more harm-inflicting to one gender, we asked participants to evaluate the negative effects of the program for those experiencing the instrumental harm (men or women, depending on the condition).

### Measures

#### Acceptance of Instrumental Harm

We crafted six items based on the IH subscale of The Oxford Utilitarianism Scale (Kahane et al., 2018): (1) The program is worth pursuing, despite the fact that more [wo]men found it insensitive and offensive; (2) despite these drawbacks, I would support this program in my own organization; (3) despite its drawbacks, this program could be a valuable tool for improving employees' work experiences in my organization; (4) despite its drawbacks, this program is worth adopting; (5) the drawbacks of this program outweigh its benefits, so it should be stopped (reverse coded); and (6) this research program contributes to the greater good of our society.^3^ Participants indicated their agreement on scales from 1 (*strongly disagree*) to 7 (*strongly agree*). Scores were averaged to form an IH acceptance composite (*α* = 0.92).

#### Perceptions of Instrumental Harm Severity

We assessed the possibility that participants might view the negative externalities of the program to be more harmful to men/women. To rule out this possibility, participants reported their agreement with the statement: “This study has a negative effect on men/women” on a scale from 1 (*strongly disagree*) to 7 (*strongly agree*). This statement always reflected the gender who experienced worse outcomes as a result of the workplace intervention (e.g., men in the male IH condition). An independent samples *t* test revealed these perceptions did not differ across conditions, *t*(155) = 0.18, *p* = .861, 95% CI = [− 0.47, 0.56], indicating participants judged the intervention’s instrumental harm as equally severe for both male and female employees across conditions.

## Results

### Hypothesis Testing

Per our pre-registration, we conducted an independent samples *t* test comparing the IH endorsement composite across conditions. Supporting our primary hypotheses, participants were significantly more likely to accept IH when the recipients of harm were men (*M* = 4.51, SD = 1.43) than women (*M* = 3.94, SD = *0.1*6), *t*(155) = 2.44, *p* = .016, 95% CI [0.11, 1.01], *d* = 0.39.^4^

**Fig. 1:**
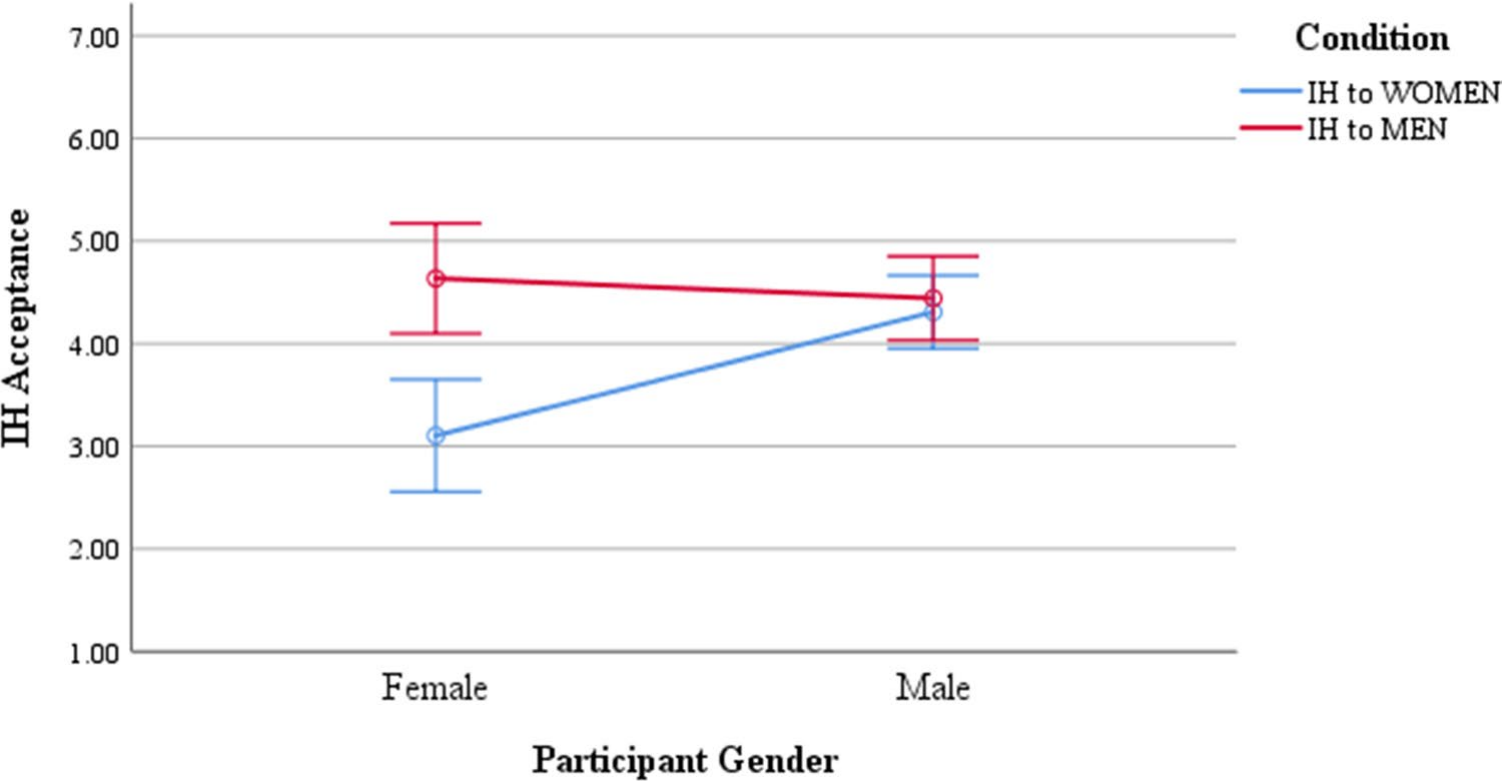
Participant gender interacts with IH recipient gender to predict instrumental harm acceptance. *Note*. Error bars represent ± 2 SEs

To test Hypothesis 2, we conducted a 2 (participant gender) X 2 (IH recipient gender) between-subjects ANOVA.^5^ Results indicated: (1) a main effect of condition, *F*(1, 151) = 12.60, *p* < .001, partial ɳ^2^ = 0.07; the program was more acceptable when IH recipients were men, rather than women (*M*_men_ = 4.51, SD_men_ = 1.39; *M*_women_ = 3.94, SD_women_ = 1.44); (2) a significant main effect of participant gender, *F*(1, 151) = 4.65, *p* = .033, partial *ɳ*^2^ = 0.03, whereby female participants were less likely to accept IH than male participants (*M*_female_ = 3.88, SD_female _= 1.61; *M*_male_ = 4.36, SD_male_ = 1.33); and (3) a significant interaction between the two factors, *F*(1, 151) = 8.88, *p* = .003, partial *ɳ*^2^ = 0.06. Supporting Hypothesis 2, post hoc tests revealed the interaction was driven by female participants’ lower acceptance of IH to women than men, whereas male participants did not show this bias (see Fig. 1).

We adjusted confidence intervals (CI) to 99% to account for multiple group comparisons. Female participants were less likely to endorse IH if it was borne by female (*M* = 3.10, SE = 0.27) than male employees (*M* = 4.64, SE = 0.27, *p* = .001, 99% CI = [− 2.60, − 0.46]). Male participants, on the other hand, did not differentially support the program based on the harmed individuals’ gender, *p* = .616. Female participants’ endorsement of IH to women was significantly lower than male participants’ endorsement of IH to other men (*M* = 4.44, SE = 0.20), *p* = .002, 99% CI = [− 2.34, − 0.17] and male participants’ endorsement of IH to women (*M* = 4.31, SE = 0.17), *p* = .004, 99% CI = [− 2.16, − 0.09]. There were no significant nor marginally significant differences between the means of the other three data points, indicating men showed no such gender bias.

## Discussion

Study 1 found support for Hypothesis 1 in revealing that participants were significantly more willing to accept IH when men suffered the instrumental harm compared to when women did. Indeed, participants were more willing to let men bear the negative externalities of the intervention, despite perceiving the negative costs as equally harmful to men and women. Importantly, these effects were driven by participant gender. Female participants evaluated a beneficial program reducing toxic workplace behaviors as more acceptable when the program inflicted IH onto men versus women, whereas male participants showed no such bias (i.e., supporting Hypothesis 2). However, Study 1 was not without its drawbacks. Although the scenario described general instances of mistreatment unrelated to sexual harassment, the organizational context may have nonetheless evoked associations with highly prevalent and salient contemporary issues (e.g., #MeToo). This particular organization context might have contributed to female participants’ lower tolerance of IH to women (who are presumably more often targets of workplace sexual harassment). Study 2 therefore sought to replicate these findings using a broader array of contexts.

## Study 2: A Constructive Replication Across Multiple Contexts

Study 2 used a mixed between- and within-subjects design. All participants evaluated five scenarios describing the efficacy of various interventions (within-subject aspect); for each of the five scenarios, participants were assigned at random to read that the treatment either benefitted women but carried for men (or vice versa). Study 2 also sought to account for individual differences that could influence the pattern of findings: (1) baseline levels of sacrificial harm endorsement, (2) egalitarianism, and (3) attitudes toward feminism.

## Method

### Participants

Based on Study 1’s main effect of *d* = 0.39 (or *f* = 0.2), G*Power indicated we would need at least 120 participants to detect a similar effect at 80% power with a repeated-measures design. To be conservative, we recruited 300 participants through Amazon’s Mechanical Turk. Recruitment, payment, and communicated study purpose were the same as specified in Study 1. After eliminating those who failed the attention check, our final sample comprised 233 individuals (51% men), with a mean age of 36.5 years (SD = 11.6).

### Procedure

Five scenarios covered a range of domains relevant to both men and women: chronic pain management, education, nutrition, psychological well-being, and sexually transmitted infections. All participants evaluated all five vignettes in randomized order. Within each scenario, we experimentally manipulated the gender of the group experiencing benefits versus harms. Thus, participants were randomly assigned to a gender condition separately for each of the five intervention scenarios. This design allowed us to assess both within-person and between-person variance in instrumental harm acceptance as a function of recipient gender, enhancing sensitivity to detect hypothesized effects. Such a design also helped ensure effects were not limited to a singular narrow context, such as in Study 1.

### Measures

#### Acceptance of Instrumental Harm

Following each intervention, participants indicated the extent to which they endorsed the program. We adapted four of Study 1’s dependent measures to apply to broader contexts: (1) despite its drawbacks, this treatment is still worth pursuing; (2) the costs of this treatment outweigh the benefits, so it should be discontinued (reverse-scored); and (3) I support adopting the treatment if it meant everyone (male or female) would have to use it; and (4) this treatment is valuable to society. Responses were reported on 7-point scales (1 = *strongly disagree*, 7 = *strongly agree*) and were averaged to form an IH acceptance composite (*α* = 0.76).

#### Control Variables

#### Baseline Sacrifice Endorsement

We measured participants’ general openness toward sacrifices using a series of sacrificial dilemmas to ensure results were driven by the IH recipient’s gender, rather than participants’ baseline endorsement of sacrificial harm. We selected three high-conflict moral dilemmas of comparable ratings (above 5.0, indicating the dilemma required a substantial sacrifice of either harm or death to another individual) from Koenigs et al. (2007). See Stimulus Materials for full wording. For each of the three cases, participants indicated whether they would take a particular action (e.g., would you throw this person overboard in order to save the lives of the remaining passengers?). Responses were coded such that 0 = *sacrificial harm rejection*, 1 = *sacrificial harm acceptance*, and these were summed to form a composite.

#### Feminism

We assessed participants’ feminist attitudes to examine whether this identification contributed to sensitivity toward women’s suffering. Participants completed three face-valid items using a 7-point scale (1 = *strongly disagree*, 7 = *strongly agree*): (1) I consider myself a feminist, (2) Modern feminists have gone too far (reverse coded), and (3) Women are still discriminated against in this country. Responses cohered well together and were therefore averaged to form a feminist identification composite (α = 0.82).

#### Egalitarianism

We assessed whether egalitarian ideology predicted greater concern over women’s than men’s suffering from IH, due to a positive association between social dominance orientation and utilitarian reasoning (Bostyn et al., 2016). We reverse-scored the 5-item Anti-Egalitarianism (AE-2) Scale (Sidanius et al., 2000, p. 67). Participants were asked to indicate the extent to which they favor each of the five items or principles on a scale from 1 (*strongly disagree/disapprove*) to 7 (*strongly agree/favor*) and responses were averaged to form a composite (*α* = 0.91). Sample items include equality, increased social equality, and increased economic equality.

## Results

### Hypothesis Testing

To account for the interdependent nature of participants’ responses, we analyzed the data using a series of 2-level hierarchical linear models (HLM 8; Raudenbush et al., 2019). The 4-item composite of intervention support was entered as the repeated dependent measure at Level 1. Gender condition was dummy coded; 0 = *men experienced IH* (i.e., women benefitted) and 1 = *women experienced IH* (i.e., men benefitted) and entered as the repeated Level 1 predictor. Level 2 accounted for between-person variance. By entering between-person variables at the Level 2 intercept, we could examine whether the main effect of the manipulation held accounting for these individual differences (e.g., participant gender, egalitarianism). When between-person variables were also entered as level 2 moderators of the Level 1 gender manipulation, we could examine whether they moderated the effect of gender condition. Level 2 variables were treated as random effects. Because participants were randomly assigned to a gender condition for each intervention separately, they each saw different numbers of male versus female-harming treatments. To account for this, participants’ average gender condition exposure was controlled at the Level 2 intercept.

Supporting Hypothesis 1, the gender manipulation significantly predicted endorsement for the interventions, *b* = -0.36, *SE* = 0.09, *t*(232) =  − 4.12, *p* < 0.001, *r* = 0.26. Participants more strongly supported interventions that helped women at the cost of men than vice versa.

A secondary model examined whether participant gender (dummy coded at Level 2) moderated the gender manipulation to test Hypothesis 2. Participant gender significantly interacted with the gender manipulation, *b* = 0.40, *SE* = 0.17, *t*(229) = 2.43, *p* = .016, *r* = .16. Female participants significantly preferred treatments benefiting women at the cost of men, *b* = -0.54, *SE* = 0.11, *t*(229) = − 4.79, *p* < .001, *r* = .30, whereas male participants did not show a significant gender bias in their treatment support, *b* = -0.14, *SE* = 0.13, *t*(229) = − 1.07, *p* = .287, *r* = .07. In line with Study 1’s results, Hypothesis 2 was again supported.

The main effect of the condition remained virtually unchanged controlling for participants’ endorsement of sacrificial harm in non-gendered contexts at the Level 2 intercept, *b* =  − 0.36, *SE* = 0.09, *t*(232) = − 4.12, *p* < .001, *r* = .26. Likewise, this main effect of condition remained significant after accounting for participants’ baseline sacrificial harm endorsement, egalitarianism, and feminist identification simultaneously at the Level 2 intercept, *b* = − 0.36, *SE* = 0.09, *t*(232) = − 4.12, *p* < .001, *r* = .26.

### Exploratory Analyses

In addition to providing direct tests of our two hypotheses, we conducted exploratory moderation analyses involving egalitarianism, feminist attitudes, and baseline harm endorsement. A third model examined whether egalitarianism interacted with the gender manipulation by entering participants’ uncentred standardized egalitarianism scores into Level 2 as a moderator of gender condition. Indeed, egalitarian endorsement significantly moderated the effect of the gender manipulation, *b* = − 0.30, *SE* = 0.08, *t*(231) = − 3.62, *p* < .001, *r* = .23. Participants who more strongly endorsed egalitarianism were more supportive of female- versus male-benefitting interventions, *b* = − 0.36, *SE* = 0.08, *t*(231) = − 4.54, *p* < .001, *r* = .29. A similar model examined the effect of participants’ feminist endorsement by entering participants’ uncentred standardized feminism scores into Level 2. Feminist identification significantly moderated the effect of the gender manipulation, *b* = − 0.24, *SE* = 0.06, *t*(231) = − 4.26, *p* < .001, *r* = .27. Participants who more strongly identified as feminists were more supportive of female- versus male-benefitting interventions, *b* = − 0.37, *SE* = 0.08, *t*(231) = -4.43, *p* < .001, *r* = .28.

Baseline sacrificial support was weakly, but not significantly, predictive of overall intervention endorsement, *b* = 0.05, *SE* = 0.06, *t*(231) = 0.78, *p* = .434, *r* = .05. Baseline sacrifice endorsement (nonsignificantly) moderated condition to predict intervention support, *b* = 0.12, *SE* = 0.08, *t*(231) = 1.43, *p* = .156,* r* = .09. That is, when women benefitted at the cost of men, baseline sacrificial endorsement was unrelated to intervention support, *b* = − 0.01, *SE* = 0.07, *t*(231) = − 0.23, *p* = .815, *r* = .02. However, when men benefitted at the cost of women, the association between baseline sacrifice endorsement and intervention support became stronger and positive, *b* = 0.10, *SE* = 0.07, *t*(231) = 1.35, *p* = .178, *r* = .09. These patterns might suggest endorsement of women’s benefit at the cost of men reflects psychological processes unrelated to baseline sacrificial tolerance, such as a general desire to advance women. However, endorsement of men’s benefit at the cost of women more strongly cohered with baseline differences in openness to sacrificial harm, raising the possibility that those who endorse utilitarian reasoning might be less likely to show gender biases in instrumental harm acceptance.

## Discussion

Study 2 constructively replicated Study 1’s findings and provided additional support for Hypothesis 1. That is, across various contexts, people more readily supported interventions that benefitted women at the cost of men than vice versa. This tendency held while controlling for baseline sacrifice endorsement, granting further support that this pattern is specific to the gender of the beneficiaries and harmed individuals, rather than the general endorsement of utilitarian principles. However, some work finds that acceptance of sacrificial harm reflects a mixture of both utilitarian reasoning and antisocial inclinations (Conway et al., 2018). Thus, it is possible that controlling for baseline harm endorsement also controlled for participants’ baseline antisocial inclinations. Supporting Hypothesis 2, female participants were more likely to endorse interventions benefitting women, but inflicting IH onto men. Male participants, on the other hand, did not show the same degree of gender bias.

Study 2 also explored the influence of ideological beliefs. Participants who more strongly endorsed egalitarianism or feminism were more supportive of interventions that benefit women at the cost of men than vice versa. These patterns suggest ideologies that emphasize the rectification of historical injustices may contribute to asymmetries in tolerance of suffering.

## Study 3: Investigation of a Boundary Condition

Studies 1 and 2 provided support that people are less willing to accept IH to women than men across a variety of interventions. Study 3 tested Hypothesis 3 by examining whether gender asymmetries in instrumental harm acceptance could be neutralized in domains in which women have been traditionally expected to sacrifice more than men: parenthood, nursing, early childhood education, and elderly care. We hypothesized that the bias to more readily accept IH to men would disappear in contexts traditionally involving female caregiving (and thus, female sacrifice), consistent with gender roles (Eagly & Wood, 1999). This study was pre-registered at https://aspredicted.org/blind.php?x=xj8j8n.

## Method

### Participants

Based on Study 2’s effect size (*r* = 0.26 or *f* = 0.27), G*Power indicated we would need at least 68 participants to detect a similar effect with a repeated-measures design at 80% power. To be conservative (especially given the anticipated smaller effect), we aimed to recruit roughly 300 participants. Recruitment, payment, and communicated study purpose were identical to previous studies. A total of 252 individuals responded to the online survey posted on MTurk. Of those, 22 failed the attention check and four did not complete the survey, and seven responses were suspected duplicates (as indicated by demographics). After these individuals were removed, the final sample comprised 225 participants (61.7% men, average age 35.1 years, SD = 11.1 years).

### Procedure

Participants evaluated five scenarios describing the efficacy of various interventions in stereotypically female contexts (e.g., nursing) benefiting the recipient group (e.g., children and the elderly), but carrying costs to the caregivers (see Appendix Table 1). Within each scenario, the gender of the harmed individuals was experimentally manipulated. Thus, participants were randomly assigned to a gender condition separately for each intervention scenario. Participants evaluated all five scenarios in randomized order. Thus, like Study 2, Study 3 employed a mixed between- and within-subjects design with an array of interventions and contexts.

#### Measures

#### Acceptance of Instrumental Harm

We used the same four items from Study 2, which were averaged to form an IH acceptance composite (*α* = 0.73).

### Control Variables

#### Feminist Identification

Study 3 employed the same 3-item measure of feminist endorsement.

#### Ideology

Participants indicated their political ideology on a 7-point Likert scale (1 = *very liberal*; 7 = *very conservative*).

## Results

### Hypothesis Testing

To account for participants’ repeated responses to the five vignettes, we again constructed two-level hierarchical models. Participants’ repeated IH acceptance composite scores were regressed onto an IH target gender dummy code (0 = *women harmed*, 1 = *men harmed*) at Level 1. In support for Hypothesis 1, we found a significant main effect of the harmed targets’ gender, *b* = 0.25, SE = 0.07, *t*(897) = 3.76, *p* < .001, *r* = .12, such that participants more strongly endorsed interventions inflicting IH onto men than women. This effect held when accounting for how many male- or female-harming interventions participants evaluated (i.e., entering total gender condition at Level 2’s intercept), *b* = 0.28, SE = 0.07, *t*(891) = 3.93, *p* < .001, *r* = .13. To examine whether participant gender moderated this effect, we entered a participant gender dummy code into Level 2. However, participant gender did not significantly moderate the main effect, *b* = 0.08, SE = 0.15, *t*(892) = 0.55, *p* = .580, indicating both male and female participants more readily supported programs inflicting instrumental harm onto men than women. In Study 3, Hypothesis 2 was not supported.

### Exploratory Analyses

Participants’ feminist identification composite scores did not significantly moderate the main effect of recipient gender, *b* = 0.04, SE = .05, *t*(896) = 0.81, *p* = .421. However, there was a marginally significant moderating effect of participants’ ideological identification (along the 7-point scale), *b* = − 0.06, SE = .03, *t*(888) = − 1.84, *p* = .066. When women were harmed, there was no effect of participants’ political ideology, such that conservative-leaning and liberal-leaning participants did not differ significantly in their IH acceptance, *b* = − 0.01, *SE* = .03, *t*(221) = − 0.05, *p* = .960. However, when men were harmed, more liberal-leaning participants more strongly supported the intervention than did more conservative-leaning participants, *b* = − 0.07, SE = .03, *t*(221) = − 1.99, *p* = .048, *r* = .13.

## Discussion

Study 3 sought to examine whether the gender bias in harm acceptance would persist across five stereotypically female contexts (e.g., nursing, grade school, and education). We found that even in contexts where women traditionally sacrificed on behalf of vulnerable individuals, both male and female participants alike more strongly endorsed interventions inflicting IH onto men than women. Our Hypothesis 1 was therefore supported in this context, whereas Hypotheses 2 and 3 were not.

Exploratory analyses revealed that unlike Study 2, participants’ feminist identification did not predict asymmetries in IH tolerance. Study 3 employed contexts whereby vulnerable individuals stood to benefit, so this pattern may suggest that all individuals (regardless of their feminist identification) are willing to accept IH to men when it could benefit vulnerable individuals. However, those more strongly endorsing feminism more readily accept IH to men when that harm benefits women (as revealed by Study 2’s findings). Study 3’s findings suggested liberal political identification exacerbated tolerance for IH on men, revealing another individual difference factor that may contribute to asymmetries in harm acceptance.

## General Discussion

The current investigation sought to examine whether people were more willing to endorse interventions when IH was borne by men than women. Our first two studies supported this premise. Importantly, however, our results showed that this asymmetry was driven primarily by women, but not men, being more likely to accept IH to men than to women across a variety of contexts (i.e., supporting Hypothesis 2). Study 3 tested a boundary condition to this gender bias in harm tolerance: stereotypically female caregiving contexts. When instrumental harm benefitted vulnerable individuals (e.g., infants, young children, sick, or the elderly), both men and women exhibited a bias in their willingness to accept IH to men versus women (i.e., supporting Hypothesis 1; not supporting Hypothesis 3). That is, contrary to what might be expected by historical gender roles (Eagly & Wood, 1999), people believed men ought to bear greater costs, even in traditionally female sacrificial domains.

### Theoretical and Practical Implications

Our findings offer four contributions. First, we extended the literature on gender and harm endorsement, which has primarily emphasized high-conflict sacrificial dilemmas involving questions of life or death (e.g., FeldmanHall et al., 2016; Skulmowski et al., 2014). The current findings revealed this gender bias persists in highly consequential, yet understudied domains: assessments of beneficial interventions carrying negative externalities across a variety of contexts: medical, psychological, educational, sexual, and caregiving. Second, we demonstrated that when evaluating interventions, female participants were more likely than male participants to accept IH borne by men than women. This pattern lends further support to the well-documented finding that women have a stronger in-group bias than men (e.g., Glick et al., 2004; Rudman & Goodwin, 2004) and are more likely to perceive one another as victims than perpetrators (Reynolds et al., 2020). This disparity suggests women may prioritize one another’s welfare over men’s in the construction or approval of social, educational, medical, and occupational interventions. If so, female policymakers might be especially wary of advancing policies or initiatives risking harm to other women, but less so when they risk harming men.

Third, we tested a boundary condition to this gender bias by investigating contexts previously unstudied in sacrificial dilemmas: stereotypically female caregiving roles. Although consideration of gender stereotypes and role congruence (Eagly & Wood, 1999) might predict a greater tolerance for female sacrifice in such contexts, men and women alike were more tolerant of IH incurred by men (versus women). These patterns suggest that although women traditionally fill and sacrifice in these roles, people may not necessarily endorse that ought to be the case. Rather, our results align with emerging evidence documenting diminished concern for men’s suffering due to a greater tendency to stereotype men as perpetrators rather than victims (Reynolds et al., 2020).

Fourth, our findings identified individual-level factors that contribute to asymmetries in harm tolerance. Namely, Studies 2 and 3 revealed that individuals more strongly endorsing egalitarian, feminist, or liberal ideologies exhibited greater disparities in their acceptance of instrumental harm, such that they more readily tolerated instrumental harm borne by men. These patterns suggest those most concerned about rectify- ing historical injustices might most ardently oppose explora- tory interventions potentially providing long-term benefits to women.

### Limitations, Emerging Questions, and Future Directions

Although the current investigation has its strengths (e.g., consistent results across varied contexts, within and between-person designs, diverse beneficiaries, pre-registrations), it is not without limitations. First, future investigations might profit, for example, from examining contexts that explicitly signal one’s willingness to sacrifice on behalf of others (e.g., voluntary military service or blood donation) to determine the generalizability of these patterns. Second, our conclusions are limited by our reliance on American MTurk and CloudResearch users. Thus, our results might not generalize to other contexts and cultures. Indeed, changes in stereotypes over time (Charlesworth & Banaji, 2022), and cultural differences in norms surrounding masculinity and femininity might shift beliefs about the value of IH incurred by men versus women (see Glick et al., 2004 for a cross-cultural comparison of attitudes toward men and women). Examining whether the reluctance to expose women to instrumental harm emerges across cultures remains an open avenue for future work. Moreover, our data were collected during the earlier days of COVID-19, which could have influenced the composition or motivations of our samples (Arechar & Rand, 2021). Thus, replication is warranted before strong conclusions can be inferred.

Fourth, although the results of Studies 1 and 2 consistently revealed women’s gender bias in instrumental harm acceptance, their methods could not disentangle whether the bias more strongly emerged from an aversion toward harming women or a desire to benefit women. That is, because both studies pit harm to one sex against the benefit to the other, it is unclear which more strongly contributed to these findings. That Study 3’s female participants (along with male) more readily tolerated men’s (versus women’s) suffering in contexts benefitting vulnerable individuals (rather than women) suggests the possibility Studies 1 and 2’s results reflected women’s greater aversion to harming fellow women, rather than a motivation to benefit them per se. Nonetheless, future research might examine interventions whereby only one sex is benefitted or harmed to adjudicate the relative contribution of these two factors.

Altogether, our findings point to potentially consequential implications for laypeople’s perceptions of exploratory interventions and programs. The asymmetry we documented may place disparate pressures on researchers and policymakers to intervene experimentally on men’s versus women’s afflictions in ways that minimize instrumental harm to women. The biases uncovered here suggest the possibility that women were excluded historically from exploratory research due to an aversion toward inflicting instrumental harm onto women, such as in medicine (Holdcroft, 2007). This ultimately proved costly to women, as men’s overrepresentation in medical research yielded treatments more effective among men than women (Holdcroft, 2007). Thus, although such an aversion may have benefitted women in the short term because women were spared incidental harm imposed by risky experiments, in the long run, experimentation on men unearthed medical and safety advancements better suited for male bodies. Experimental examinations and interventions carry both costs and benefits. If, as our results suggest, people are less willing to accept instrumental harm befalling women, women might lose out on the long-term benefits of such experimental endeavors.

Throughout history, countless male lives have been sacrificed on the battlefield, ostensibly to promote the greater good (Baumeister, 2010). Our findings suggest that these sentiments persist beyond the field of combat. For many people, accepting instrumental harm to men is perceived as worth the cost to advance other social aims. We invite researchers to further investigate how individuals appraise the value of suffering and whether those appraisals differ across target characteristics. A deeper understanding of the biases embedded in such calculations may minimize the unforeseen and unintended consequences of those preferences, thereby reducing harm to men and women alike.

### Electronic supplementary material

Below is the link to the electronic supplementary material.

Supplementary file1 (DOCX 119 KB)

## Figures and Tables

**Table 1:**
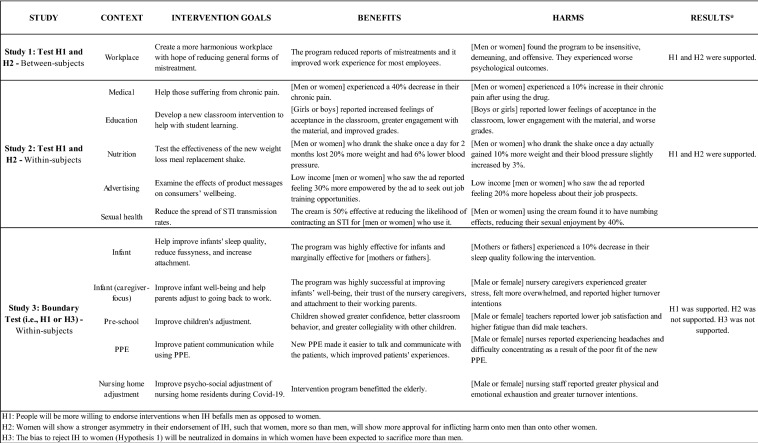
Complete studies and contexts

## Data Availability

OSF Data Link https://osf.io/dc4b5/?view_only=7fd015b384574eee8346fcaae569aa44

## Appendix 1

See Table 1.

## Footnotes

1 We use the term interventions to consider a variety of programs aimed to advance human welfare. Examples include conducting scientific experiments and implementing organizational or educational programs intended to produce desired outcomes.

2 *r* = .21 is the average effect size of social psychology research (Richard et al., 2003). We selected it due to the lack of prior reference for our effect.

3 We included three additional items, and we report those in SOM due to space and fit. Importantly, our conclusions remain unchanged regardless of whether we use the 6-item scale (1-factor) vs. 9-item scale (2-factor). Our data and syntax reproduce those results.

4 We considered the possibility that our results could be influenced by our attention check exclusion approach. Additional results from both complete (i.e., all responses) and the most conservative responses are available in SOM. In summary, findings remained largely unchanged regardless of our exclusion approach.

5 Our Hypothesis 2 testing was not pre-registered for Study 1 and should therefore be viewed as “exploratory.” The reason for that is the evolution of our theory. Although we were initially interested only in the main effect, a new member of our team offered theoretical insight which encouraged us to place greater emphasis on the interplay between the gender of participants and actors.
