# Supplementary material for: Worth the Risk? Greater Acceptance of Instrumental Harm Befalling Men than Women
Source: Arch Sex Behav. 2023 Mar 17;52(6):2433–45. doi: 10.1007/s10508-023-02571-0 (PMC10022566; doi:10.1007/s10508-023-02571-0)
Supplement: Supplementary file 1 — Supplementary file1 (DOCX 119 KB) [file 10508_2023_2571_MOESM1_ESM.docx]

**Worth the Risk? Greater Acceptance of Instrumental Harm Befalling Men than Women**

**SUPPLEMENTARY ONLINE MATERIALS**

**This document includes all materials used in our studies. We provide the following:**

1. **Stimulus materials (pages 2 – 10).** We note our materials as they were officially worded. We also explain in greater detail how we designed our interventions descriptions and we provide relevant references.
2. **Supplementary analyses (pages 11+).** We provide supplementary analyses of variables included for exploratory purposes.

The commonalities among all studies are that the informed consent was presented to participants at the very beginning of the study (i.e., prior to stimulus materials).

**STIMULUS MATERIALS**

**STUDY 1 MATERIALS:**

**ASSESSMENT OF ORGANIZATIONAL INTERVENTIONS**

**Holistic well-being workplace program description:**

A research team from a large business school recently concluded the first stage of a comprehensive study where they investigated the effectiveness of a 4-week long holistic well-being program. The program can be implemented in any industry, but it is particularly designed for workplaces with toxic behaviors where employees report harassment, incivility, petty comments, discrimination, and overall poor behavior from others.

Researchers predicted that exposing employees to this structured holistic well-being program can lead to more harmonious workplaces. It incorporates elements of mindfulness, cognitive behavioral therapy, resilience, and it increases awareness of harassment, discrimination, and incivility against men and women. The goal of the program is to help employees become more aware of their behavior and be more mindful of their coworkers.

**Core results:**

In comparison to a control group (i.e., another successful initiative), the program reduced reports of harassment, incivility, damaging gossip, and discrimination by 36% and it improved work experience for most employees.

Overall, most participants found the program to be beneficial. However, there were some troubling differences between men and women.

Men [women] found the program to be insensitive, demeaning and punitive towards men [women], and at times simply offensive.

Those men [women] experienced feelings of lower psychological safety, higher work-related stress symptoms, lower psychological well-being, and higher intentions to leave.

**Comprehension check questions:**

*Instructions to participants*: Please indicate whether each of these statements is true or false:

1. The program reduced reports of harassment, incivility, damaging gossip, and discrimination by 36%.
2. Most found the program to be highly beneficial.
3. The program improved working experience for most employees.
4. Men found the program to be insensitive and offensive towards men [women].
5. The program focused on reducing workplace mistreatment.

All statements were designed to be true. If participants missed one or more true/false questions, they were asked to read the case one more time and they were given the same questions.

**Manipulation check to ensure our responses are not due to different inferences of harm:**

- This study has a negative effect on men [women]; 1 (*strongly disagree*) to 7 (*strongly agree*)

**Acceptance of Instrumental Harm (Main DV):**

*Instructions to participants*: Your organization has an opportunity to implement this program. As you are answering the following questions, keep in mind the program's costs and benefits:

Participants noted their responses on a scale from 1 (*strongly disagree*) to 7 (*strongly agree*)

1. The program is worth pursuing, despite the fact that more men [women] found it insensitive and offensive.
2. Despite these drawbacks, I would support this program in my own organization.
3. Despite its drawbacks, this program could be a valuable tool for improving employees' work experiences in my organization.
4. Despite its drawbacks, this program is worth adopting.
5. The drawbacks of this program outweigh its benefits, so it should be stopped (*reversely coded).*
6. This research contributes to the greater good of our society.

**Attention check question:**

- Please select 'agree' for this question.

**Alternative and exploratory indicators:**

Results are reported in *Supplementary Analyses* file (starting with page 11 of this document):

1. I would support my own taxpayer money funding additional research that would make sure the program is not demeaning or offensive to men [women].
2. The research methodology of this program is rigorous.
3. If I found out that my taxpayer dollars funded this research, I would be supportive.

Unless otherwise noted, all studies contained the following demographic questions:

- Identification
  - 1 = Male, 0 = Female, 9 = Gender diverse or other
- Age
- Ethnic background (not analyzed)
- Political ideology (not analyzed)
  - 1 = Extremely liberal
  - 2 = Liberal
  - 3 = Somewhat liberal
  - 4 = Moderate
  - 5 = Somewhat conservative
  - 6 = Conservative
  - 7 = Extremely conservative
- Level of education (not analyzed)
  - 1 = Less than high school
  - 2 = High School / GED
  - 3 = Some College
  - 4 = 2-year College Degree
  - 5 = 4-year College Degree
  - 6 = Professional Degree (e.g., Law or Medicine)
  - 7 = Master’s Degree
  - 8 = Doctoral Degree

**STUDY 2 MATERIALS:**

**A CONSTRUCTIVE REPLICATION ACROSS MULTIPLE CONTEXTS**

*The following assessment was presented first (i.e., before the experimental manipulations)*

**Control variable: Baseline (gender-neutral) assessments of utilitarian judgment**

1. You are part of a group of ecologists who live in a remote stretch of jungle. The entire group, which includes eight children, has been taken hostage by a group of paramilitary terrorists. One of the terrorists takes a liking to you. He informs you that his leader intends to kill you and the rest of the hostages the following morning. He is willing to help you and the children escape, but as an act of good faith he wants you to kill one of your fellow hostages whom he does not like. If you refuse his offer all the hostages including the children and yourself will die. If you accept his offer then the others will die in the morning but you and the eight children will escape.
   1. *Would you kill one of your fellow hostages in order to escape from the terrorists and save the lives of the eight children?*

Yes No

**Study 2 responses**: Yes (68.0%) No (32.0%)

1. You are on a cruise ship when there is a fire on board, and the ship has to be abandoned. The lifeboats are carrying many more people than they were designed to carry. The lifeboat you’re in is sitting dangerously low in the water—a few inches lower and it will sink. The seas start to get rough, and the boat begins to fill with water. If nothing is done it will sink before the rescue boats arrive and everyone on board will die. However, there is an injured person who will not survive in any case. If you throw that person overboard the boat will stay afloat and the remaining passengers will be saved.
   1. *Would you throw this person overboard in order to save the lives of the remaining passengers?*

Yes No

**Study 2 responses**: Yes (72.0%) No (28.0%)

1. You are negotiating with a powerful and determined terrorist who is about to set off a bomb in a crowded area. Your one advantage is that you have his teenage son in your custody. There is only one thing that you can do to stop him from detonating his bomb, which will kill thousands of people if detonated. To stop him, you must contact him over the satellite hook-up that he has established and, in front of the camera, break one of his son’s arms and then threaten to break the other one if he does not give himself up.
   1. *Would you break the terrorist’s son’s arm in order to prevent the terrorist from killing thousands of people with his bomb?*

Yes No

**Study 2 responses**: Yes (85.6%) No (14.4%)

**Manipulations: Within-subject Test of Intervention Programs**

All participants received all five intervention scenarios. Gender of the treatment beneficiaries and instrumental harm recipients was randomized within each of the five scenarios. There were two conditions: 1) Women benefit, but men encounter risks, or 2) Men benefit, but women encounter risks.

**Interventions scenarios:**

1. Researchers have been testing a new medical treatment to help those suffering from chronic pain. The drug is ingested orally and operates on the brain’s pain pathways. Preliminary results find that the effects of the drug depend on whether the patient is male or female. By 6 weeks of treatment, men experienced a 40% decrease in their chronic pain. However, women experienced a 10% increase in their chronic pain after using the drug.
2. Education experts have developed a new classroom intervention to help with student learning. Over the course of the semester, students give brief speeches to their classmates about how the subject material applies to their own everyday lives. When the experts looked at large scale results of this intervention, they found that following the speeches, girls reported increased feelings of acceptance in the classroom, greater engagement with the material, and improved grades. Strangely enough, however, boys who performed this speech exercise reported lower feelings of acceptance in the classroom, lower engagement with the material, and worse grades.
3. Nutrition scientists are testing out a new weight loss meal replacement shake. The shake is intended to replace one meal a day. The scientists examined the effects of the shake on weight loss and found an interesting pattern. Women who drank the shake once a day for 2 months, tended to lose 20% more weight and have 6% lower blood pressure. However, men who drank the shake once a day actually gained 10% more weight and their blood pressure slightly increased by 3%.
4. Advertisers have examined the effect of product messages on consumers’ wellbeing. One large scale company is testing out the effects of a new ad campaign. This ad documents all of the major hardships associated with being low income in modern society. They’ve presented this ad to many individuals across the country. Low income women who saw the ad reported feeling 30% more empowered by the ad to seek out job training opportunities. Low income men who saw the ad, however, reported feeling 20% more hopeless about their job prospects. Follow-up studies using behavior confirmed these patterns. Women who saw the ad were more likely to complete a job training program, but men were more likely to drop out of the program.
5. The spread of sexually transmitted infections has become a huge concern to the broad public. Scientists have discovered a topical cream that has been shown to be somewhat effective in reducing STI transmission rates. The cream can be applied to either partner’s genital region. The cream is in its second phase of clinical testing and the results have been mixed. The cream is 50% effective at reducing the likelihood of contracting an STI for women who use it. The cream did not reduce men’s odds of contracting STIs however. Moreover, men exposed to the cream found it to have numbing effects, reducing their sexual enjoyment by 40%.

**Acceptance of Instrumental Harm:**

Immediately after reading each of the interventions, participants were asked to indicate the extent to which they endorse the program. Specifically, they were asked to indicate the extent of their agreement with each of the following items on a scale from 1 (*strongly disagree*) to 7 (*strongly agree*).

1. Despite its drawbacks, this treatment is still worth pursuing.
2. The costs of this treatment outweigh the benefits, so it should be discontinued (*reversely coded*)
3. This treatment is valuable to society.
4. I support adopting the treatment if it meant everyone (male or female) would have to use it.

**Attention check question:**

- Please select 'agree' for this question.

**Control and additional variables:**

**Attitudes towards feminism (written for the purposes of the study)**

1= *strongly disagree*, 7 = *strongly agree*

1. I consider myself a feminist
2. Modern feminists have gone too far (reverse coded)
3. Women are still discriminated in this country.

**Social dominance orientation (Sidanius et al., 2000, p. 67); AE-2 Scale**

Below are a series of statements with which you may either agree or disagree. For each statement or a phrase, please indicate the degree of your agreement/disagreement by checking the appropriate number from 1 (*strongly disagree or strongly disapprove*) to (*strongly agree or strongly approve*). Once again, remember that your first responses are usually the most accurate.

1. Equality.
2. This country would be better off if we cared less about how equal all people were.
3. If people were treated more equally we would have fewer problems in the country.
4. Increased social equality.
5. Increased economic equality.

**STUDY 3 MATERIALS:**

**INVESTIGATION OF A BOUNDARY CONDITION**

**Manipulations:**

***Within-subject Test of Intervention Programs in Stereotypically Female Caregiving Contexts***

All participants received all five intervention scenarios. Gender of the treatment beneficiaries and instrumental harm recipients was randomized within each of the five scenarios. There were two conditions: 1) Men encounter risks (but the caregiving group benefits), or 2) Women encounter risks (but the caregiving group benefits).

**Intervention scenarios:**

1. Researchers tested a behavioural-educational sleep intervention program to help infants and first-time parents sleep better. The sleep intervention group received prenatal education and guidance about their own and their infant’s sleep patterns during the first three months of the baby’s life. The control group received no treatment. Results showed that infants’ sleep quality was much improved in the intervention group. Infants in this group slept more soundly, their crying and fussiness decreased, and they showed greater engagement with the family members. However, the program was ineffective for fathers. Although mothers experienced a slight improvement in their sleep quality, fathers experienced a 10% decrease in their sleep quality following the intervention.
2. A large school district tested an intervention to improve children’s adjustment. The rigorous one-year program focused on 4- and 5-year olds. It required teachers and their aides maintain weekly contact with parents, allocate time for one-on-one interactions with each child, and make two home visits per year. The program was an absolute success for the young children. A year after the program, children who participated showed greater confidence in the activities, exhibited better classroom behavior, and greater collegiality with other children. Following the program, however, female teachers reported lower job satisfaction and higher fatigue than did male teachers.
3. A major city hospital tested the effectiveness of new personal protective equipment (PPE) for its nursing staff working with Covid-19 patients. The new PPE was designed to address the well-documented shortcomings of previous equipment. It also aimed to improve verbal communication for both nurses and their patients. Overall, the new PPE made it easier to talk and communicate with the patients, which improved patients' experiences. However, the hospital observed that the new PPE fit men’s faces better than women’s. Women described the new PPE as ‘uncomfortable’ and ‘restrictive’ when working with 12-hour shifts. Female nurses reported experiencing headaches and difficulty concentrating as a result of the poor fit.
4. A nursing home tested a new intervention program to improve psycho-social adjustment of its residents during Covid-19 times. The program involved exercise, online engagement with children from local elementary schools, and individualized attention to assigned nursing home residents. The intervention program benefited the elderly and it improved their overall psychological health and social adjustment. Unexpectedly, however, there were gender differences in staff experiences. Male nursing home staff reported positive experiences with the program. Female nursing home staff who participated in the program reported greater physical and emotional exhaustion and greater turnover intentions.
5. A nursing home tested a new intervention program to improve psycho-social adjustment of its residents during Covid-19 times. The program involved exercise, online engagement with children from local elementary schools, and individualized attention to assigned nursing home residents. The intervention program benefited the elderly and it improved their overall psychological health and social adjustment. Unexpectedly, however, there were gender differences in staff experiences. Male nursing home staff reported positive experiences with the program. Female nursing home staff who participated in the program reported greater physical and emotional exhaustion and greater turnover intentions.

**Acceptance of Instrumental Harm:**

Immediately after reading each of the interventions, participants were asked to indicate the extent to which they endorse the program. Specifically, they were asked to indicate the extent of their agreement with each of the following items on a scale from 1 (*strongly disagree*) to 7 (*strongly agree*).

1. Despite its drawbacks, this treatment is still worth pursuing.
2. The costs of this treatment outweigh the benefits, so it should be discontinued (*reversely coded*)
3. This treatment is valuable to society.
4. I support adopting the treatment if it meant everyone (male or female) would have to use it.

**Attention check question:**

- Please select 'agree' for this question.

**Control and additional variables:**

**Sex**

1. Male
2. Female
3. Other

**Age**

**Political affiliation (1 = very liberal; 7 = very conservative)**

**Education**

1. Some high school
2. High school graduate
3. Some college
4. Bachelor’s degree
5. Graduate degree

**Ethnicity (Not analyzed)**

1. White
2. Asian
3. Latin/Hispanic
4. Black
5. Middle Eastern
6. Mixed
7. The following:_____________

**Attitudes towards Feminism**

1= *strongly disagree*, 7 = *strongly agree*

1. I consider myself a feminist
2. Modern feminists have gone too far (reverse coded)
3. Women are still discriminated in this country.

**SUPPLEMENTARY ANALYSES**

**STUDY 1 ADDITIONAL ANALYSES:**

**ASSESSMENT OF ORGANIZATIONAL INTERVENTIONS**

**Footnote 4 results: Main effects using all responses.** We analyzed the data using all responses (i.e., without eliminating responses from participants who failed our attention check questions). Supporting our primary hypothesis, participants were significantly more likely to accept IH when the recipients of harm were men (*M* = 4.62, *SD* = 1.30) than women (*M* = 4.10, *SD* = 1.30), *t*(199) = 2.79, *p* = .006, 95% CI [.16, .92], *d* = .39.

**Exploratory factor analysis results: 6-item Instrumental Harm Scale**. The EFA statistics indicated that the 6 items load on a single factor (Costello & Osborne, 2005; Fabrigar et al., 1999). We based this conclusion on inspection of the eigenvalues, which showed that only one eigenvalue exceeded 1.0. Visual inspection of the scree plot revealed that the break in the plot occurred below one data point. The first factor accounted for 72.61% of the total variance. All factor loadings surpassed the recommended threshold of .50 (Costello & Osborne, 2005).

**Assessment of alternative indicators.** The following three items were assessed as potential alternative indicators of instrumental harm acceptance. They were not included in the full scale because EFA results suggested that the three items load on a different factor. Therefore, we report those results separately.

1. **Taxpayer I**: I would support my own taxpayer money funding additional research that would make sure the program is not demeaning or offensive to women.
2. **Taxpayer II**: If I found out that my taxpayer dollars funded this research, I would be supportive.
3. **Methodological rigor**: The research methodology of this program is rigorous.

Participants indicated their agreement on a scale from 1 (*strongly disagree*) to 7 (*strongly agree*).

**SOM Table 1**

*Study 1: Descriptive Statistics and Correlations Between the Assessed Variables*


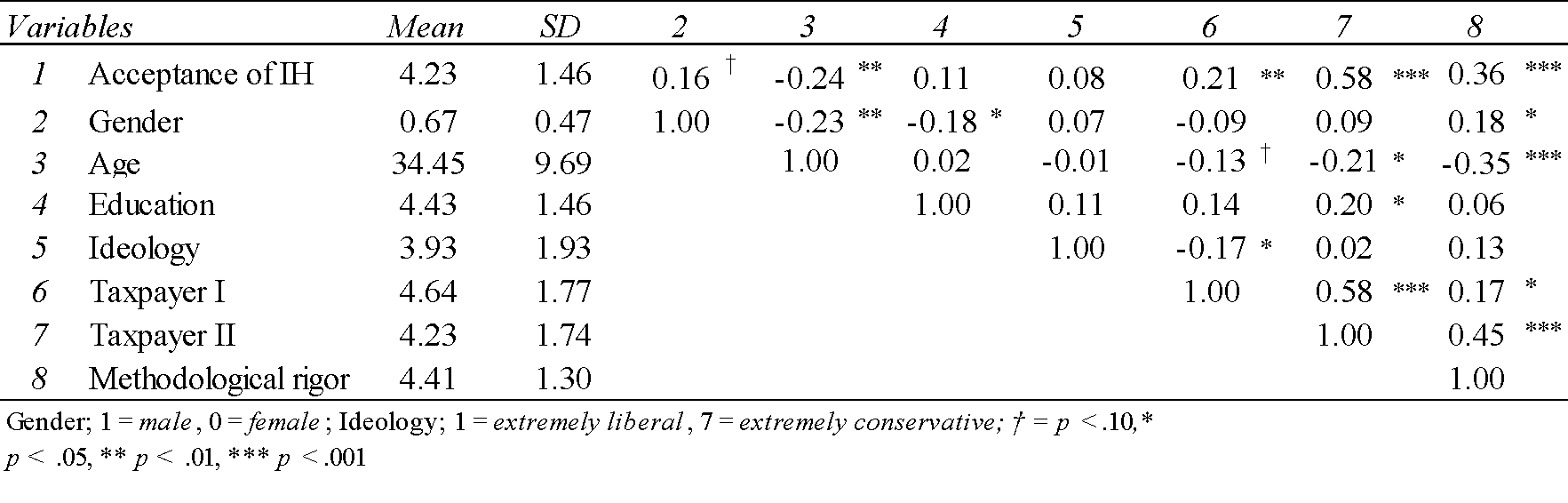


**Alternative indicators: Results**. The following table serves as a complement to Study 1 between-group comparison. For transparency purposes, we report findings using Acceptance to IH as a four-item measure (as noted on AsPredicted.org). An independent samples *t* test comparing responses to three alternative variables between the conditions yields no significant differences in participants’ assessments of methodology and acceptance of own taxpayer money being used for additional research. However, participants indicated they would be more accepting of their taxpayer dollars funding this research, if the IH recipients were men rather than women (Taxpayer II variable).

**SOM Table 2**

*Study 1: Descriptive Statistics and T Test Results for Alternative Indicators*


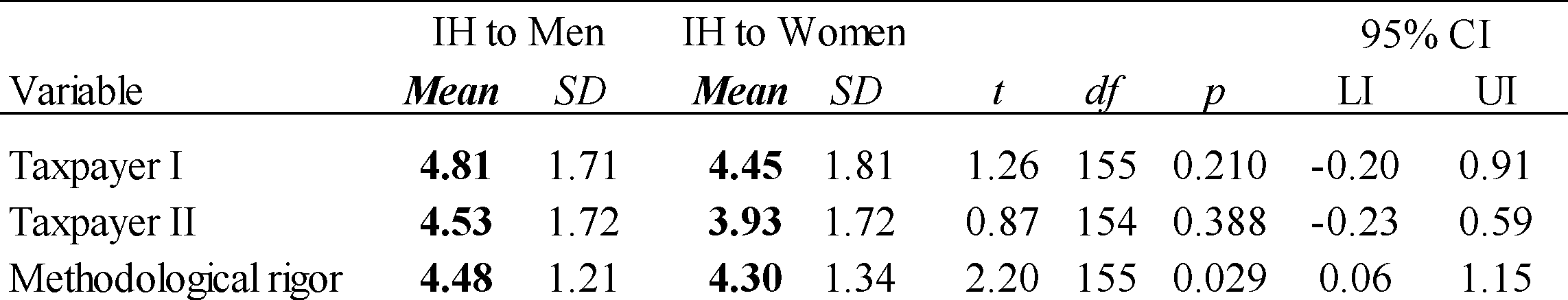


**STUDY 2 ADDITIONAL ANALYSES:**

**A CONSTRUCTIVE REPLICATION ACROSS MULTIPLE CONTEXTS**

**Study 2 Methods**

***Attention Check: Additional Descriptions and Results***

Our pre-registration specified that we would retain responses from participants who passed all attention checks. However, we observed that such a conservative strategy reduced our sample size to 65 and 75 participants per male- and female-focused condition respectively, which was below our targeted 80 participants per condition. For the purposes of analysis reporting, we therefore retained participants who failed only 1 attention check. These exclusion criteria resulted in our final sample size of 160 participants. However, to ensure that our conclusions are not influenced by our exclusion tactic, we note any deviations from these reported results that would compromise the integrity of our findings.

In order to examine whether these results were influenced by our exclusion tactic, we conducted the same analyses using all responses, and using only perfect responses (i.e., no missed attention checks). We observed significant effect of the condition even in the most restricted sample, *t*(135) = 2.49, *p* = .014, 95% CI = [.13, 1.13]. This analysis yielded the least supportive results (i.e., the highest *p*-value), and even those findings were significant and supportive of our Hypothesis 1. Using the full sample (*N* = 200) yielded *p* = .005.

***Acceptance of Instrumental Harm: Additional Measures Descriptions***

Our pre-registration specified that we would nine items in total (three of which were exploratory indicators). Exploratory factor analysis suggested that 6 items noted above yielded one factor (information and alternative analyses using 4-item IH scale are provided in Stimulus Materials). Three exploratory alternative indicators are: 1) The research methodology of this program is rigorous; 2) I would support my own taxpayer money funding additional research that would make sure the program is not demeaning or offensive to men/women; and 3) If I found out that my taxpayer dollars funded this research, I would be supportive.

**Study 2 Results**

***Supplementary Analyses***

Descriptive statistics and correlations are presented in SOM Table 1.

**SOM Table 3**

*Study 2: Descriptive Statistics and Correlations Between IH, Gender and Control Variables (Sacrificial Harm Endorsement, Feminism, and Egalitarianism)*

| *Variables* | | *Mean* | *SD* | *2* |  | *3* |  | *4* |  | *5* |  |  |
| --- | --- | --- | --- | --- | --- | --- | --- | --- | --- | --- | --- | --- |
| *1* | Acceptance of IH | 4.07 | 0.91 | 0.80 |  | -0.05 |  | 0.04 |  | -0.05 |  |  |
| *2* | Participant Gender | 0.50 | 0.50 |  |  | -0.11 | ^†^ | -0.12 | ^†^ | 0.09 |  |  |
| *3* | Sacrificial Harm Endorsement | 2.24 | 0.96 |  |  |  |  | -0.01 |  | -0.03 |  |  |
| *4* | Feminism | 4.36 | 1.69 |  |  |  |  |  |  | 0.65 | *** |  |
| *5* | Egalitarianism | 5.54 | 1.46 |  |  |  |  |  |  |  |  |  |
|  |  |  |  |  |  |  |  |  |  |  |  |  |
| Gender; 1 = *male*, 0 = *female*; *† = p* < .10*,* p <* .05, ** *p <* .01, *** *p* < .001 | | | | | | | | | | | | |
